# Alternative Protein Sources in Poultry and Pig Nutrition—A Review

**DOI:** 10.1111/jpn.70047

**Published:** 2026-02-10

**Authors:** Lukáš Čumplík, Dana Zálešáková, Jakub Novotný, Lenka Kudlová, Leoš Pavlata, Ondřej Šťastník

**Affiliations:** ^1^ Department of Animal Nutrition and Forage Production, Faculty of AgriSciences Mendel University in Brno Brno Czech Republic

**Keywords:** insects, lupine, nettle, peas, seaweed, single‐cell protein

## Abstract

Proteins are an essential nutrient for the viability of all animals, enabling organisms to grow, regenerate and defend themselves against pathogenic organisms. Soybean and soybean‐based materials are commonly used to supplement protein in animal nutrition. However, soybeans are among the most expensive ingredients in feed mixtures, and the expansion of their planting areas has led to rainforest deforestation. Soybean cultivation is also associated with soil degradation, extensive use of chemicals and fossil fuels and global warming. This is partly because soybeans are grown primarily in specific regions of the world and therefore must be transported over long distances. This is a costly process that has a significant negative impact on the environment and does not meet sustainability requirements. At the same time, global population growth is increasing protein consumption, putting even more pressure on the environment. It is therefore necessary to look for different alternative feeds that could replace soybeans or at least reduce their dominance in the feed supply. Several studies mention new non‐traditional feeds that could help solve this problem. This review provides an overview of available information on alternative protein sources and their use in poultry and pig nutrition, thereby assisting in their selection. The characteristics of selected alternative protein feeds (such as insects, algae, lupine, peas, faba beans, single‐cell protein and nettle) and their effects on the performance or health parameters of the animals mentioned are described below.

## 1 Introduction

Currently, there is a need to explore new alternative protein sources for animal nutrition. At the same time, the world's growing human population is increasing the pressure on food supplies and, therefore, on the availability of proteins. The search for feeds that will supply these nutrients while meeting sustainability requirements is essential (Siddiqui et al. 2023). The main source of protein used in the nutrition of non‐ruminant animals is soybeans and products made from soya. However, this reliance is problematic from an environmental perspective. Importing it from South American countries is relatively expensive and also involves significant consumption of fossil fuels (Pexas et al. 2023).

Thornton et al. (2023) stated that climate change is expected to reduce livestock production over the next 20 years, although this will also depend on other factors influencing future agricultural systems. One of these factors is the environmental impact of agriculture and animal production. Feeds such as insects and insect products or single‐cell protein supply the necessary amount of protein with their composition, and their production is not dependent on the soil. There is also no need to cut down rainforests for their production, thereby reducing animal populations in affected areas. All of this should contribute to lower greenhouse gas emissions and a reduced carbon footprint (Hefferon et al. 2023; Z. Zhang et al. 2024).

When setting feed rations, it is necessary to take into account not only the nutritional quality of the feed and its environmental impact, but also the financial cost, and, as noted by Thornton et al. (2023), it is also very important to consider the national and regional context. Instead of using feed transported over long distances, with significant consumption of fossil fuels, it should be gradually replaced by locally produced feed ingredients (Te Pas et al. 2021). This would reduce the pressure to adjust feed production in South America, which would have a positive effect on soil quality and ecosystems (Pexas et al. 2023).

This review includes several alternative sources that may help reduce the need for soybean meal: insects, seaweed, lupin, peas, faba beans, nettles and single‐cell protein. Table 1 presents a comparison of the crude protein, crude fat, crude fibre and antinutrient content of these alternative protein feeds relative to soybean meal. The collected data on the effect of including insects, seaweed, lupins, peas and faba beans, nettle and single‐cell proteins in feed mixtures for non‐ruminants are presented in Tables 2, 3, 4, 5, 6 for greater clarity.

**Table 1:** Comparison of chemical composition and antinutrients in selected protein feeds (data presented in dry matter, %).

Protein feeds	Crude protein	Crude fat	Crude fibre	Antinutrients	References
Soybean meal	48.4	3.5	4.1	Phytates, tannins, trypsin inhibitor, oligosaccharides	Smit et al. (2021), Adeyemo and Onilude (2013)
Mealworm (larvae)	53.3	29.4	6.2	Benzoquinone compounds, accumulation of heavy metals, toxins produced by the exocrine gland	Šťastník et al. (2021b), Hong et al. (2020)
Black soldier fly (larvae)	43.0	34.1	10.4	Accumulation of heavy metals, allergens, phytate, tannin, haemagglutinin, trypsin inhibitor	Mahmud et al. (2020), Bessa et al. (2021), Afam‐Ibezim et al. (2025)
Housefly (larvae)	15.3	1.2	33.5	Microorganisms contained	Hussein et al. (2017), Niode et al. (2022)
Silkworm (pupae)	35.9	32.1	2.1	Accumulation of heavy metals, lignans, alkaloids	Akande et al. (2020), Köhler et al. (2019), de la Luz Sánchez‐Estrada et al. (2024)
Earthworm meal	41.4	9.2	1.8	Accumulation of heavy metals	Janković et al. (2020), Kavle et al. (2023)
*Ulva lactuca*	18.6	1.4	8.8	Glucosinolates, saponins, tannins	Bajad et al. (2024), Macoveiu et al. (2025)
*Spirulina*	68.2	5.0	x	Phytate, trypsin inhibition	Lugarà et al. (2022), Tibbetts et al. (2023)
*Chlorella*	46.0	9.3	1.6	Saponin, phytate, tannins	Coelho et al. (2020), Chen et al. (2022)
Yellow lupine (Tarper v.)	46.3	5.4	19.0	Trypsin inhibitors, lectins, alkaloids, phytate	Sobotka et al. (2016), Kaczmarek et al. (2016), Bessada et al. (2019), Olkowski (2018)
White lupine	39.7	11.2	14.7	Trypsin inhibitors, lectins, alkaloids	David et al. (2024), Bessada et al. (2019)
Blue lupine	35.8	7.4	19.5	Trypsin inhibitors, lectins, alkaloids	David et al. (2024), Bessada et al. (2019)
Stinging nettle (leaf powder)	33.8	3.6	9.1	Phytate, oxalate, saponins	Adhikari et al. (2016), Bessada et al. (2019)
Field peas	26.6	2.0	11.4	Trypsin inhibitors, tannins, saponins, oxalates, phytic acid, isoflavones	David et al. (2024), Dong et al. (2025)
Faba bean (Fabelle v.)	32.2	0.1	7.2	Tannins, phytase, vicin, convicin	Smit et al. (2021), Milczarek (2024)
Single‐cell protein (bacteria)	50‐65	1‐3	x	High nucleic acid content—allergic reactions, toxic effects^a^	Li et al. (2024), Alves et al. (2025)
Single‐cell protein (fungi)	30‐45	2‐8	x	High nucleic acid content—allergic reactions, toxic effects^a^	Li et al. (2024), Alves et al. (2025)
Single‐cell protein (*Saccharomyces cerevisiae*)	53.4	1.92	x	High nucleic acid content—allergic reactions, toxic effects^a^	Agboola et al. (2021), Alves et al. (2025)

*Note:* x ‐ data not found.

^a^ Negative effects may occur more with long‐term feeding; feeding is recommended for short‐lived animals.

**Table 2:** Comparison of the inclusion of insects in feed mixtures and their effect on production parameters and animal health.

Protein feeds	Animal category	Proportion in feed mixture (%)	Impact on production	Impact on health	References
Black soldier fly (larvae)	Hens	1−3	No effects on body weight gain, egg yield, egg weight or egg mass, lightening of the yolk		Lokaewmanee et al. (2023)
		1−3	Increased egg production in late laying period		Yan et al. (2023)
		20	No negative impact on performance		Tahamtani et al. (2021)
	Weaned piglets	2.5 + astaxanthin	Reduced the susceptibility to fat oxidation without negatively affecting production parameters		Szczepanik et al. (2023)
		19		Effect on growth intensity or digestive tract health	Håkenåsen et al. (2021)
	Fattening pigs	14	Higher carcass weights and weight gains		Chia et al. (2021)
Mealworm (larvae)	Hens	3	Improved colour of yolk, composition of the yolk, specifically the α‐linolenic acid content		Ko, Choi, et al. (2020)
		5		No negative impact	Šťastník et al. (2021a)
	Broilers	3	Positive effect on body weight and feed conversion ratio, no negative effect on the quality of thigh meat		Sedgh‐Gooya et al. (2022); Štastník et al. (2021b)
		10	Positively influenced the carcass yield		Vasilopoulos et al. (2023)
	Weaned piglets	3	No effects on the performance		Ko, Kim, et al. (2020)
		10	No effects on the performance	Effect on the population of microorganisms and fermentation activity in the caecum	Meyer, Gessner, Braune, et al. (2020); Meyer, Gessner, Maheshwari, et al. (2020)

**Table 3:** Comparison of the inclusion of seaweeds in feed mixtures and their effect on production parameters and animal health.

Protein feeds	Animal category	Proportion in feed mixture (%)	Impact on production	Impact on health	References
*Chlorella*	Hens	1	Increased eggshell		Esakkimuthu et al. (2024)
	Fattening pigs	5	No negative effect on growth or on meat quality	Positively influenced the content of desirable n‐3 fatty acids	Coelho et al. (2020)
*Chondrus crispus* and *Ascophyllum nodosum*	Hens	3 and 1.5		Help cope with heat stress	Borzouie et al. (2020)
Nhlane et al.		4	No negative effect on meat quality	Increased the weight of the caecum and decreased the weight of the proventriculus and duodenum	Nhlane et al. (2021)
*Ulva spp*.		16		Effect on fat metabolism, reduced cholesterol levels in the blood serum	Dewi et al. (2023)
	Broilers	1		A reduced concentration of total lipids in the blood serum	Abudabos et al. (2013)
		3	Increased breast yield without affecting weight gain or feed conversion		Abudabos et al. (2013)
*Ascophyllum*	Piglets	1		Positively influenced the activity of antioxidant enzymes	M. Costa et al. (2021)
*Laminaria digitata*	Fattening pigs	10		Reduced the content of n‐6 fatty acids in relation to n‐3, increased iodine	Moroney et al. (2015)

**Table 4:** Comparison of the inclusion of lupines in feed mixtures and their effect on production parameters and animal health.

Protein feeds	Animal category	Proportion in feed mixture (%)	Impact on production	Impact on health	References
Yellow lupine	Broilers	20	No negative effect on weight gain and body weight		Pietras et al. (2021)
White lupine	Broilers	32	No negative effect on final broiler weight, feed conversion	Lower mortality	Straková et al. (2024)
	Male fattening pigs	12	Positive effect on growth and carcass quality		Moore et al. (2021)
Blue lupine	Hens	25	Negative impact on egg weight, positively influences the yolk colour (increased levels of lutein)		Kowalska et al. (2020); Kowalska et al. (2021)
	Piglets	30	Negative effect on body weight gain		Tuśnio et al. (2020)
	Weaned piglets	20	No negative effect on the growth		Zaworska‐Zakrzewska et al. (2020)
	Fattening pigs	17.5		Increase levels of bioactive substances in meat, reduce levels of total protein, liver enzymes or magnesium in the blood	Sońta et al. (2022); Sońta et al. (2020)

**Table 5:** Comparison of the inclusion of peas and faba beans in feed mixtures and their effect on production parameters and animal health.

Protein feeds	Animal category	Proportion in feed mixture (%)	Impact on production	Impact on health	References
Peas	Broilers	20	No negative effect on the performance		Nalle et al. (2011)
		35	No negative effect on the performance		Diaz et al. (2006)
		40	Reduced lightness and yellowness of the muscle, decreased n‐6/n‐3 polyunsaturated fatty acid ratio in chicken thighs		Laudadio and Tufarelli (2010)
		48	No negative effect on the performance		Dotas et al. (2014)
	Fattening pigs	17.5		Reduced creatine, cholesterol or urea levels in blood	Sońta et al. (2020)
		30	No effect on growth		Babatunde et al. (2021)
	Guinea fowl	18	Decreased n‐6/n‐3 polyunsaturated fatty acid ratio, cholesterol and saturated fatty acid levels in the meat		Laudadio et al. (2012)
Faba beans	Broilers	10	No negative effect on the performance		Kim et al. (2023)
		16	Without a significant decrease in performance		Koivunen et al. (2014)
		20	Without a significant decrease in performance		Nalle et al. (2010)
	Fattening pigs	40	No negative effect on the performance		Babatunde et al. (2021)

**Table 6:** Comparison of the inclusion of nettle and single‐cell proteins in feed mixtures and their effect on production parameters and animal health.

Protein feeds	Animal category	Proportion in feed mixture (%)	Impact on production	Impact on health	References
Nettle	Broilers	0.15	Improved growth parameters, feed conversion ratio, and breast yield		Gao et al. (2024); Milosevic et al. (2021)
		0.5	Increase in the content of Zn, Se and Fe in the thigh muscles and an increase in n‐3 fatty acids		Stanišić et al. (2023)
		4		Decreased levels of cortisol and cholesterol	Mirsaiidi Farahani and Hosseinian (2022)
	Quails	6		Reduction of triglycerides and cholesterol in blood serum	Moula et al. (2019)
	Fattening pigs	1	Positive effect on protein deposition in the body		Szewczyk et al. (2006)
Single‐cell protein	Broilers	Below 1	No effect on the feed conversion ratio		Khan et al. (2024)
		Up to 2	Negative effect on weight gain, conversion or feed intake		Bhunia et al. (2023)
		10	Negative effect on weight gain and feed conversion ratio		Hombegowda et al. (2021)

## 2 Feed Overview

### 2.1 Insects

Insects are one of the possible alternatives as protein sources. There are a number of different types of insects that can be used for these purposes (Thornton et al. 2023). Commonly used insects include mealworms (*Tenebrio molitor*), black soldier flies (*Hermetia illucens*), earthworms (*Lumbricina*), house flies (*Musca domestica*) and silkworms (*Bombyx mori*), particularly in the nutrition of non‐ruminant animals (Elahi et al. 2022). The nutritional composition of insects is strongly influenced by their diet (Thornton et al. 2023). In addition, the proportion of other essential amino acids may be higher in insects compared to plant‐based feed (Elahi et al. 2022). The main disadvantage of feeding mealworms, *T. molitor*, is their high cost, as their production is relatively inefficient due to low production. High costs are associated with the substantial infrastructure demands, as well as the energy and feed consumption required for production (Siddiqui et al. 2023). The resulting nutritional quality of insect‐based feed, such as mealworms, is strongly influenced by the quality of breeding, feed and processing technologies (Slimen et al. 2023). To obtain insect protein, insects must undergo several processing steps, including pre‐cleaning (e.g., drying or sieving), defatting, protein solubilization (using acidic, basic, salt and aqueous solutions), regeneration of the supernatant, protein purification and final purification (Queiroz et al. 2023). When using mealworms, product safety should also be considered, as the larvae may contain toxins produced by the exocrine glands. The deposition of heavy metals in the insect's body is another problem (Hong et al. 2020). Another disadvantage of insects as feed components is their limited large‐scale production capacity compared to other alternative protein sources (Siddiqui et al. 2023). Nevertheless, insects used as animal feed may contribute to sustainability and a reduction in global warming potential (Vauterin et al. 2021).

Black soldier fly larvae contain high proportions of essential amino acids, such as alanine, leucine and valine. Housefly larvae are also high in essential amino acids such as valine or arginine (Shah et al. 2022). However, the high protein content in insect products is not the only factor to consider. It is also closely related to protein digestibility, which is substantially lower in insects compared to sources like soy or legumes (Siddiqui et al. 2023). For example, protein digestibility in black soldier fly larvae is approximately 51% (Slimen et al. 2023).

Consumer acceptance of products derived from insect‐fed animals is also an important factor. Spartano and Grasso (2021) reported that 72% of respondents in Great Britain would be willing to try such products, and 87% would be willing to purchase them if prices met expectations. Consumers were willing to pay up to 18% more for eggs produced by insect‐fed hens. In contrast, research conducted in Hungary showed that respondents rated meat from insect‐fed animals significantly lower than meat from free‐range systems. It should be noted that free‐range animals may consume insects that have not undergone any quality control (Szendrő et al. 2020).

#### 2.1.1 Insects in Poultry Nutrition

According to a study by Tahamtani et al. (2021), the inclusion of 20% live black soldier fly larvae did not negatively affect the performance of laying hens. Even Yan et al. (2023) reported that with increasing content of black soldier fly larvae in the diet (from 1% to 3%), egg production increased late in the laying period and affected the population of microorganisms in the caecum. They also stated that replacing up to 75% of fish meal with black soldier fly larvae meal did not change carcass quality. An increase in egg production has not been confirmed by several studies (Lokaewmanee et al. 2023; Ko, Kim, et al. 2020). The addition of *T. molitor* larvae meal positively affects not only the colour of the yolk but also the composition of the yolk, specifically the α‐linolenic acid content (Ko, Choi, et al. 2020). However, the inclusion of live black soldier fly larvae in the diets for laying hens resulted in a lightening of the yolk colour associated with increasing insect content in the feed (Lokaewmanee et al. 2023). Eggshell thickness, egg shape and Haugh units were not affected by the inclusion of *T. molitor* (Rahmawati et al. 2022).

Several studies also monitored the influence of the inclusion of insects in feed mixtures on the content of n‐3 fatty acids. Rahmawati et al. (2022) reported that the addition of up to 5% *T. molitor* increases the content of these health‐promoting substances. Feeding live black soldier fly larvae did not increase the n‐3 fatty acid content of eggs (Lokaewmanee et al. 2023). However, it may affect the composition of the microflora in the caecum (Yan et al. 2023).

Including up to 5% mealworm meal did not negatively affect feed quality and can therefore be safely used in laying hen diets (Šťastník et al. 2021a). *T. molitor* larvae meal fed at 2.5% of the diet had a positive effect on body weight in broiler chickens and, at the same time, improved feed conversion in the initial stage of fattening (Sedgh‐Gooya et al. 2022). Whole larvae inclusion (5%–10%) positively affected carcass yield and meat quality (Vasilopoulos et al. 2023). Šťastník et al. (2021b) reported that 2%–5% mealworm meal had no negative effect on thigh meat quality.

#### 2.1.2 Insects in Pig Nutrition

According to Lestingi (2024), the use of insects or insect products in pig nutrition is more economically demanding and therefore more problematic than in poultry nutrition. The production of forage insects is low compared to the need for pig nutrition, and at the same time, the cost is high, which reduces the usability of insects as a component in pig diets. In particular, a considerable number of experiments have been carried out with the inclusion of insects in diets for fattening weaned pigs (Hong and Kim 2022). Weaned piglets fed diets containing *T. molitor* larvae or meal showed no differences in live weight, feed intake or average daily gain (Ko, Kim, et al. 2020; JonasLevi and Martinez 2017; Meyer, Gessner, Braune, et al. 2020). Similarly, diets containing *Alphitobius diaperinus* did not affect weight gain or feed intake, although increased dorsal fat deposition was observed with higher inclusion levels (Müller Richli et al. 2023). Research by Håkenåsen et al. (2021) shows that up to 19.06% full‐fat black soldier fly larvae meal can be included in the diet for weaned piglets with a small effect on growth intensity or digestive tract health. Replacing 50%–100% of fish meal with black soldier fly larvae meal resulted in higher carcass weights and growth rates in fattening pigs (Chia et al. 2021).

Veldkamp and Vernooij (2021) state that the digestibility of amino acids in insect meals (full‐fat/partially defatted black soldier fly larvae meal or housefly meal) is similar to that of soybean or fish meal. At the same time, however, they point out the high variability of digestibility and growth parameters, which depend on several factors, such as the composition of the feed ration, the chemical composition of the insects and also the concept of the experiment itself.

Szczepanik et al. (2023) administered a mixture of *H. illucens* larvae meal and astaxanthin to weaned piglets in order to monitor the growth and health status of the piglets after weaning, which is a risky period for them. The inclusion of 2.5% of these components reduced the susceptibility to fat oxidation without negatively affecting production parameters. Meyer, Gessner, Maheshwari, et al. (2020) observed that 10% *T. molitor* meal increased proteobacteria and branched‐chain fatty acid concentrations in the caecum.

### 2.2 Seaweed

Seaweed can also be used in the nutrition of non‐ruminant animals. Approximately 12,000 species have been identified, and algae are classified as macroalgae or microalgae based on size (Thornton et al. 2023; Ibrahim et al. 2023; Kulshreshtha et al. 2020). Macroalgae are further divided into brown (*Phaeophyceae*), green (*Chlorophyceae*) or red (*Rhodophyceae*) algae (M. Costa et al. 2021) and can reach a length of several metres. They are a rich source of proteins, fatty acids (such as docosahexaenoic acid and eicosapentaenoic acid), vitamins, astaxanthin and carotenoids, and can have a positive effect on meat quality (Thornton et al. 2023; Ibrahim et al. 2023). Microalgae contain bioactive compounds such as alginate, fucoxanthin or laminarin. With their composition, they can contribute to improving the health of the digestive tract of animals through their immunomodulating or antimicrobial effect (Dewi et al. 2024). Different types of seaweed contain different amounts of protein. Some species, such as *Arthrospira*, can contain up to 60%−70% proteins (Amorim et al. 2021). Microalgae are also sources of fat‐soluble vitamins (A, D, E, K) and water‐soluble vitamins (C, B12) (Ibrahim et al. 2023). Carotenoids contained in seaweed, especially xanthophylls, reduce the risk of cancer or inflammation in the body. The group of xanthophylls includes, for example, lutein or zeaxanthin, which are mainly contained in *Spirulina spp*., *Chlorella spp*. or *Scenedesmus spp*. (A. G. Pereira et al. 2021). Just mentioned *Spirulina*, according to the study by Pootthachaya et al. (2023), contains a high amount of protein, and the protein digestibility is relatively high. Spirulina also contains a high amount of essential amino acids such as lysine, threonine and tryptophan. The mentioned properties prove the suitability of *Spirulina* as a possible feed for poultry. Thornton et al. (2023) state in their study that the problem with this type of feed is its inefficient production. It's connected with high economic demands, mainly the costs of cultivation and harvesting (Amorim et al. 2021). In order to use seaweed in animal diets, large quantities of seaweed need to be grown, and then the excess water removed so that it can be used for further processing. It is also worth noting the high NaCl content, which must be reduced for the health of the animals. The necessary technological procedures for this treatment also increase the price of feed (Dewi et al. 2024). After processing and various treatments, the required dose of seaweed is incorporated into feed mixtures, which can be further pelleted (Esakkimuthu et al. 2024). Despite all the mentioned shortcomings, it can be stated that the cultivation of seaweed represents a gentle way of producing protein feeds (M. M. Costa et al. 2024).

#### 2.2.1 Seaweed in Poultry Nutrition

Esakkimuthu et al. (2024) reported that the addition of 1% *Chlorella* seaweed increased eggshell thickness by 6 μm. This is also supported by the study of Rahmatnejad et al. (2021), who stated that feeding *Spirulina platensis* and brown seaweed increased the shell thickness of quail. In addition, the size of the resorption surface in the gut was positively affected, resulting in better utilization of dietary nutrients. The addition of seaweed can also help animals cope with heat stress, particularly the addition of 3% *Chondrus crispus* and 0.5% *Ascophyllum nodosum* (Borzouie et al. 2020). *Sargassum binderi* can affect fat metabolism in laying hens, such as LDL content, which was reduced in serum. The inclusion of 16% of these brown algae in the feed mixtures also significantly reduced cholesterol levels in the blood serum (Dewi et al. 2023). Nhlane et al. (2021) reported that feeding green seaweed, specifically *Ulva* spp., affected the weight of digestive tract organs in broilers. The inclusion of the algae increased the weight of the caecum and, conversely, decreased the weight of the proventriculus and duodenum.

The addition of green seaweed to broiler diets increased daily feed intake as well as weight gain (Nhlane et al. 2020). An experiment conducted by Nhlane et al. (2021) showed no negative effect of up to 3.5% of green algae on final broiler meat quality. A 3% proportion of the green seaweed *Ulva lactuca* increased breast yield without affecting weight gain or feed conversion. Even with the inclusion of 1% *U. lactuca*, a reduced concentration of total lipids in the blood serum was monitored (Abudabos et al. 2013).

However, the high NaCl content in seaweed can have a negative effect on poultry, which can cause health problems. An increase in dietary NaCl is associated with increased water intake and, consequently, the threat of diarrhoea (Dewi et al. 2024). However, the inclusion of seaweed in the feed diets of broilers, that is, poultry, can be considered as one alternative to supplement protein, but also other beneficial substances such as vitamins, minerals or bioactive substances. Despite the higher NaCl content, no negative effects on the health or production of broilers have been reported (Nhlane et al. 2020; Ibrahim et al. 2023).

#### 2.2.2 Seaweed in Pig Nutrition

M. Costa et al. (2021) stated in their study that *Ascophyllum*, one of the most common and well‐studied algae, has a positive effect on the health of the animal's intestines and thus on their production parameters, thanks to its probiotic properties. The proportion of *Ascophyllum* in the diet (up to 1%) for piglets positively influenced the activity of antioxidant enzymes, such as superoxide dismutase, which helps the body cope with oxidative stress. There was also a positive effect on lipid metabolism and crude protein digestibility (Czech et al. 2024). Dierick et al. (2009) add that Ascophyllum in piglet diets also increases iodine levels in several tissues. When 5% of the algae *Chlorella vulgaris* was added to fattening pigs, there was no reduction in growth intensity or a negative effect on meat quality. On the contrary, the addition positively influenced the content of desirable n‐3 fatty acids (Coelho et al. 2020). As reported by D. M. Ribeiro et al. (2021), the use of seaweed as a feed additive can influence not only the sensory properties and composition of meat but also its colour. This is also confirmed by Michalak et al. (2020), who monitored the effect of seaweed enriched with ions, which can also be used as an alternative to inorganic salts. When the algae *Enteromorpha* spp. were enriched, zinc and copper ions affected the sensory properties of the meat. The meat was darker, with a higher protein content and a lower water content, as indicated by a higher dripping. The ability of algae to influence the composition of pork is also supported by the study of D. Ribeiro et al. (2022). Moroney et al. (2015) added that 10% of *Laminaria digitata* algae increased the content of some elements, such as iodine, and also reduced the content of n‐6 fatty acids in relation to n‐3. *L. digitata* contains the polysaccharides laminarin and fucoidan. After their extraction and addition to the diet of pigs for 3 weeks at doses of up to 900 mg/kg, reduced fat oxidation and saturated fatty acid levels in the meat were recorded.

Lugarà et al. (2022) investigated the effect of including 20 g of *Spirulina* in the diet of pregnant/lactating sows on the offspring. *Spirulina* tended to affect the growth of the offspring, but the effect was negative in male offspring.

### 2.3 Lupine

Lupine is a promising alternative for replacing or reducing soybean use in feed rations due to its relatively high protein content (Mikulski et al. 2014). From the point of view of the composition of the protein structure, albumins and globulins predominate (Boukid and Pasqualone 2022). The present globulins include β‐conglutin (content of 44%−45% of globulins), α‐conglutin (content of 35%−37% of globulins), δ‐conglutin (content of 10%−12% of all proteins) and γ‐conglutin (content of 4%−5% of all proteins) (Duranti et al. 1981, 2008). Glutamic acid is the most abundant amino acid, followed by arginine and aspartic acid. Lupine contains higher levels of glutamic acid and arginine than soybeans and peas, but lower methionine and total essential amino acids (Boukid and Pasqualone 2022).

More than 200 lupine species exist, with yellow (*Lupinus luteus*), white (*Lupinus albus*) and narrow‐leaved (*Lupinus angustifolius*) being the most common (Olkowski 2018). Nalle et al. (2012) add that for the purposes of growing lupine with a higher protein content, two other varieties are used, namely *Lupinus mutabilis* and *Lupinus polyphillus*. The three most commonly cultivated varieties mentioned are of European origin. However, in 2015, Oceania accounted for 75.3% of lupine production, while Europe contributed only 17.6% of the total production (Lucas et al. 2015). This state corresponds to the current situation. In 2022, the largest producer was Australia, which produced 957,000 tons of lupins, more than half of the world's production (Helgilibrary 2023).

In terms of production in the European Union, 370,155,960 tonnes of lupines were grown on 218,970 ha in 2023. This is a small decrease in production compared to 2022; however, in the long term, lupine production in the European Union is gradually increasing (FAOSTAT 2025).

One of the disadvantages of using lupine in animal nutrition is the presence of anti‐nutritional factors, especially high levels of toxic alkaloids and phytic acid, which significantly reduce the availability of phosphorus and other elements to animal organisms (Olkowski 2018). Lupanine, sparteine, nutalline, angustifolin or oxylupanine can be mentioned as examples of the alkaloids contained (Cortés‐Avendaño et al. 2020). A. Pereira, Ramos, et al. (2022) list protease inhibitors, lectins and raffinose family oligosaccharides as other anti‐nutritional substances, in addition to alkaloids and phytates. The maximum legal content of alkaloids in lupine dry matter is set at 0.2 g/kg (Cortés‐Avendaño et al. 2020). The level of alkaloids must be monitored because feeding diets with a high content of these substances may lead to health problems, manifested by reduced feed intake, hypersalivation, vomiting, tremors or convulsions and, in the most extreme cases, death of animals (Boukid and Pasqualone 2022; Boschin et al. 2022). However, through selective breeding, the levels of these antinutrients decreased, and toxicity was practically eliminated (Olkowski 2018). By breeding, the alkaloid content is reduced below 1%, although it may still vary. Additionally, there are technological procedures that can be achieved by reducing the alkaloid content, for example, soaking the seed in water and then boiling it (Boukid and Pasqualone 2022). Villacrés et al. (2020) reported in their study that the reduction of bitter taste caused by alkaloids was more effective when lupine was boiled in a sodium chloride solution compared to boiling it in a plain aqueous solution. Another potential treatment for feed can be fermentation. Fermentation of narrow‐leaved lupine increases crude protein, crude fibre and ash content, while simultaneously reducing the levels of anti‐nutritional substances in the seeds (Zaworska‐Zakrzewska et al. 2020; Kasprowicz‐Potocka et al. 2021). However, this adjustment can be economically disadvantageous, as noted by Zaworska‐Zakrzewska et al. (2020), due to the low profitability associated with enhancing the nutritional value of seeds.

#### 2.3.1 Lupine in Poultry Nutrition

Pietras et al. (2021) examined the differences in performance between broilers fed a 33% soybean meal ration and a 60% soybean meal replacement with lupine, between 21 and 42 days of age. No differences were found in weight gain and body weight. Also, no qualitative changes were observed in the carcass. Straková et al. (2024) reported that even 100% replacement of soybean meal with white lupin had no negative effect on final broiler weight, feed conversion and even replacement had a positive effect on animal health. Replacing soybean with lupine should not negatively affect egg quality—egg weight and Haugh unit (Kowalska et al. 2020). However, a high proportion of lupine, such as 25% or more, can have a negative impact on egg weight (Kowalska et al. 2020, 2021). This is also confirmed by Krawczyk et al. (2024), who stated that the 28% inclusion of legumes (peas, faba beans, lupins) does not impair egg‐laying performance but may lead to the deterioration of egg quality parameters. Regarding the effect on blood biochemical parameters, replacing soybean with lupin leads to a reduction in total protein and triacylglycerides in laying hens (Straková et al. 2021) and cholesterol in broilers (Viveros et al. 2007). Additionally, feeding lupine instead of soybean to laying hens may reduce cholesterol and saturated fatty acids while increasing monounsaturated and polyunsaturated fatty acids in egg yolk (Timová et al. 2020; Kowalska et al. 2020). Moreover, the proportion of lupine in the feed ration positively influences the yolk colour of the laid eggs (Kowalska et al. 2020). The yolk colour is partially influenced by carotenoids such as lutein (Kowalska et al. 2021), which is an important factor for consumers (Kowalska et al. 2020).

#### 2.3.2 Lupine in Pig Nutrition

The use of lupine as a substitute for soybeans, that is, extracted soybean meal, has been tested, similarly to its use in poultry, also in pigs. Zaworska‐Zakrzewska et al. (2020) state that the use of lupine (both raw and fermented) as a replacement for 50% of extracted soybean meal does not lead to a reduction in production indicators. However, piglets fed between the 35th and 46th day of age with a diet where lupine completely replaced the extracted soybean meal had lower gains than piglets in which the extracted soybean meal was only partially replaced (Tuśnio et al. 2020). The solution may be found in lupine fermentation, which improves the digestibility of proteins and some amino acids. In addition, an increase in the total number of bacteria and yeasts was achieved in pigs. When comparing narrow‐leaved lupine and yellow lupine, fermentation seems to be more effective in narrow‐leaved lupine, at least in terms of pig nutrition (Kasprowicz‐Potocka et al. 2021). Moore et al. (2021) report that a diet containing 12% white lupine is optimal for growth and carcass quality while reducing feed consumption in immunocastrated male pigs. Biochemical analyses of the blood did not suggest that the replacement of 17.5% soybean meal by narrow‐leaved lupine would have a negative impact on the health of the pigs. This is, although reduced levels of total protein, liver enzymes or magnesium were detected in the blood (Sońta et al. 2020). The inclusion of lupine in the feed ration can also improve the meat quality of pork. When compared with diets containing 5%−17.5% narrow‐leaved lupine and diets where proteins were supplemented mainly with extracted soybean meal, pigs fed this legume had a higher value of bioactive substances (Sońta et al. 2022).

There are also several studies addressing the combination of lupine with other feeds (pea, faba bean, rapeseed meal and others). Feeding a combination of yellow lupine, pea and rapeseed meal does not affect pork quality compared to the use of soybean meal. However, there may be a decrease in growth intensity and a reduction in body fat (Zmudzińska et al. 2020). However, a reduction in fat deposition may be desirable in castrated male pigs, which gain fat just after castration (Moore et al. 2021).

### 2.4 Peas and Faba Beans

During 2023, a total of 782,630 tonnes of peas were grown on an area of 141,820 ha in the Member States of the European Union (FAOSTAT 2025). Pea seeds contain 15.3% neutral detergent fibre, 8.5% acid detergent fibre, 41.3% starch and 3.1% ash. They also contain a high level of essential amino acids, particularly lysine, leucine and arginine. However, the limiting amino acid is methionine (David et al. 2024). Regarding the content of anti‐nutritional substances, trypsin inhibitors, phytic acid, chymotrypsin inhibitors, tannins, α‐amylase inhibitors and lectins are found in peas (Alonso, Grant, et al. 2000). The variety *Pisum sativum* var. *hortense* is suitable for animal nutrition because it does not contain tannins (Jezierny et al. 2010). The concentration of trypsin inhibitors can be reduced by dehulling, since they are predominantly located in the hull. Lectins are glycoproteins that cause agglutination of red blood cells, thereby negatively affecting digestion by binding to the intestinal mucosa. These undesirable properties can be reduced by heat treatment (Gatel 1994). Another feed processing method that improves nutritional value is extrusion. In addition to the reduction of the already mentioned anti‐nutritional substances, extrusion increased the digestibility of proteins, but also of starch (Alonso, Grant, et al. 2000).

Faba beans contain 22.1% neutral detergent fibre, 11.6% acid detergent fibre, 3.6% starch and 4.1% ash, as well as a high content of lysine or group B vitamins (Rahate et al. 2021; Meng et al. 2021). As for the protein structure in faba beans, albumins, globulins, prolamins and glutenins are mostly contained. Similar to peas, methionine is the limiting amino acid in faba beans (Feng et al. 2024). However, faba beans contain, like other legumes, a whole range of anti‐nutritional substances such as trypsin inhibitors, tannins, lectins, vicine, convicine or phytic acid (Rahate et al. 2021; Meng et al. 2021). A common treatment that should be used to reduce these undesirable substances is dehulling, seed soaking, sprouting, enzymatic treatment of fermentation or heat treatment (Rahate et al. 2021). Alonso, Aguirre, et al. (2000) reported that dehulling increases the protein content and, at the same time, reduces the concentration of tannins, as a significant amount is localized in the seed coat.

When comparing the content of amino acids between peas, faba beans and soy, soybeans exhibit significantly higher levels. For example, the content of lysine, an essential amino acid important for growth, is 33.1 g/kg in soybeans, 17.3 g/kg in peas and 18.4 g/kg in faba beans. Methionine is contained in peas at 2.2 g/kg, which is comparable to that in faba beans; however, the content is almost three times higher in soybeans (Jezierny et al. 2010).

#### 2.4.1 Peas and Faba Beans in Poultry Nutrition

According to a study by Nalle et al. (2011), adding 200 g/kg of peas does not negatively affect the broiler performance and can be used as a substitute for soybean meal. Diaz et al. (2006) reported that adding up to 350 g/kg of peas to feed mixtures does not reduce the growth rate of broiler chickens. The addition of peas at a rate of 400 g/kg led to reduced lightness and yellowness of the muscle. Additionally, there was a decrease in the n‐6/n‐3 polyunsaturated fatty acid ratio in chicken thighs (Laudadio and Tufarelli 2010). A reduction in the n‐6/n‐3 polyunsaturated fatty acid ratio was also observed in guinea fowl fed a diet containing 180 g/kg of peas. Furthermore, there was a decrease in cholesterol and saturated fatty acid levels in the meat, without any negative effects on growth or meat yield (Laudadio et al. 2012). Up to 480 g/kg of peas can be included in the diet at the end of the fattening period of broilers without significantly affecting production parameters. There was no observed reduction in feed intake, feed conversion or meat yield (Dotas et al. 2014).

A study by Kim et al. (2023) reported that liver polyunsaturated fatty acid levels were increased in chickens fed a diet containing faba bean compared to a control group based on corn, wheat and soybean meal. The inclusion of faba bean also reduced feed conversion. The authors of the study reported that the inclusion of 100 g/kg faba bean as a replacement for soybean meal is possible without negatively affecting performance. Reduced growth associated with reduced feed intake was observed in an experiment by Koivunen et al. (2014) with increasing faba bean content in the feed ration. They reported a suitable faba bean content of 160 g/kg in the feed mixture without significant performance degradation. Nalle et al. (2010) even report a safe proportion of up to 200 g/kg faba bean in broiler diets without significant negative effects on production parameters.

#### 2.4.2 Peas and Faba Beans in Pig Nutrition

Peas can be included in feed diets for fattened pigs up to 30% (Babatunde et al. 2021). Replacing soybean meal with peas did not affect weight gain, but in the early post‐weaning period, there was an increase in the intake of a diet containing peas. The effect of pea treatment—cold‐pelleted, steam‐pelleted or extruded field—did not affect slaughter weight or daily weight gains (Hugman et al. 2020). When comparing the content of n‐3 fatty acids in the meat of immunocastrated pigs, the meat of individuals fed a diet with a proportion of soybean meal had a higher concentration than a diet where it was partially replaced by peas (up to 40% replacement) (Argemí‐Armengol et al. 2023). Blood biochemical analyses showed that the inclusion of 17.5% peas as a replacement for soybean meal to fattened pigs reduced creatine, cholesterol and urea levels (Sońta et al. 2020). When samples taken from the *longissimus lumborum* of pigs fed these diets were compared, the peas‐fed group had lower taurine levels (Sońta et al. 2022). However, the inclusion of peas as a partial substitute for extracted soybean meal has no negative effect on the physical and chemical properties of the meat (Sońta et al. 2021).

Faba beans can be included in diets for fattening pigs up to 40% (Babatunde et al. 2021). The inclusion of peas and faba beans did not affect daily weight gain, feed conversion ratio in grower pigs (up to a proportion of 30% in the feed mixture) or carcass quality (Smith et al. 2013). Pigs fed with peas (30% of the diet) had a higher percentage of lean meat than pigs fed with faba beans (30% of the diet) (White et al. 2015).

The smell of boar meat is a relatively serious problem for consumers, due to which boars are castrated/immunocastrated. Increasing the proportion of peas and faba beans in the feed mixture (up to 30%) reduced the content of indole, one of the hormones responsible for boar odour in back fat (Smith et al. 2013).

### 2.5 Nettle

As mentioned by Bhusal et al. (2022), stinging nettle has excellent nutritional and healing properties. The protein content in nettle is around 30%, with the proportion varying from plant to plant. The higher dose is located in the leaves (roughly around 23%), in opposite to the stems, with almost half of this amount (Gao et al. 2024). Moreover, nettle contains a significant amount of minerals and vitamins, and at the same time has a strong antioxidant effect, thanks to the flavonoids. Quercetin, for example, can reduce the risk of cancer. Dried leaves have higher antioxidant activity than fresh leaves. The antioxidant capacity is due to phenolic compounds, especially hesperidin (Teixeira et al. 2023). Dhouibi et al. (2020) add that stinging nettle lowers blood pressure and blood sugar, increases insulin secretion and also lowers the LDL/HDL ratio. Another positive feature is the ability to improve the functioning of the immune system (Gao et al. 2024). Vitamin C and beta‐carotene, as a provitamin of vitamin A, are also abundant in the nettle composition (Bhusal et al. 2022). Loetscher et al. (2013) state that it is precisely the high representation of beta‐carotene, lutein and zeaxanthin that the yellowness of the yolk is higher when feeding hens diets containing stinging nettle. Different parts of the plant have different beneficial effects on the organism depending on their composition. Nettle leaves have an antihyperglycaemic, hepatoprotective or antibacterial effect, the seed oil is antimicrobial and the extract of the whole plant has a diuretic effect (Jan et al. 2017).

#### 2.5.1 Nettle in Poultry Nutrition

Gao et al. (2024) reported that the addition of 0.5−1.5 g nettle per kg of feed ration improved growth parameters in broiler chickens. Stanišić et al. (2023) showed that feeding nettle affects the fatty acid profile of chicken meat and also alters its mineral content. In their study, there was an increase in the content of Zn, Se and Fe in the thigh muscles, as well as an increase in n‐3 fatty acids in male broilers, proving that nettle as a component of feed mixtures improves meat quality. Milosevic et al. (2021) reported that nettles contain substances that support growth, feed utilization and the immune system of broilers. They further report that when nettle meal was fed at 1% and 1.5%, feed conversion was significantly reduced while feed consumption remained the same. The experiment also proved that this diet had a positive effect on breast yield. In a similar trial, a diet containing 1% of dried nettle leaves was fed. Compared with the control group, there was an increase in body weight at 42 days of age in chickens. Gao et al. (2024) reported that the addition of 6.25−25 g per kg of diet improved yolk colour.

The addition of nettle to the diet can also influence serum triglyceride and cholesterol concentrations, particularly at a 1.0% inclusion level (Milosevic et al. 2021). A reduction in blood serum triglycerides and cholesterol when feeding a diet containing 6% stinging nettle was also observed in Japanese quail (Moula et al. 2019). Another positive aspect of nettle feeding was described by Mirsaiidi Farahani and Hosseinian (2022). In their study, they recommended the addition of dicotyledonous nettle to improve the health of broilers fattened under suboptimal thermal conditions. When nettle was administered at 4%, cortisol levels, an indicator of stress, as well as cholesterol, AST, CK and ALT, decreased.

#### 2.5.2 Nettle in Pig Nutrition

The use of nettle extract at a concentration of 0.5 g/kg had a positive effect on protein deposition in the bodies of fattening pigs, compared to pigs fed a diet without nettle. Furthermore, this proportion of nettle extract did not affect the growth parameters of pigs. The addition of nettle extract had no negative effect on crude protein digestibility (Szewczyk et al. 2006). Another possible use of nettle in pig diets is in the form of an extracted mixture of various herbs. Hanczakowska et al. (2015) experimented using a mixture of nettle, sage, lemon balm and echinacea in a ratio of 0.15 g and 0.5 g per kg. The results showed an improvement in pork quality, particularly in terms of the meat's oxidative stability, as well as an increase in the content of beneficial polyunsaturated fatty acids, without negatively affecting production parameters. An extract composed of the same herbs was used in an experiment with piglets. In this case, piglets fed a diet containing 0.5 g/kg of herbal extract showed a higher growth rate compared to piglets fed a diet without the extract. Piglets fed a feed mixture with herbal extract showed an increase in intestinal villus height, which enhances the absorptive surface area for nutrient uptake (Hanczakowska and Swiatkiewicz 2012).

### 2.6 Single‐Cell Protein

As pointed out by Z. Zhang et al. (2024), single‐cell protein production is a sustainable alternative to traditional protein feed production. This sustainability is attributed to the ability to produce protein from biomass, which requires less land than the cultivation of plant‐based protein sources. Single‐cell protein can contain up to 80% protein on a dry matter basis (Gundupalli et al. 2024; A. G. Pereira, Fraga‐Corral, et al. 2022). These proteins are derived from single‐celled microorganisms, such as seaweed, yeast, bacteria or fungi, and are produced through fermentation (Gundupalli et al. 2024; Li et al. 2024). Fermentation is followed by harvesting, after which the cells are separated, and then the cells are disrupted to make the proteins more accessible. Subsequently, the material needs to be cleaned and spray‐dried (Gundupalli et al. 2024). Species such as *Methylococcus* spp. or *Methylobacterium extorquens*, for example, can be utilized for this purpose (A. G. Pereira, Fraga‐Corral, et al. 2022; Hombegowda et al. 2021). Conversely, fungi and yeast generally provide a lower protein content (only 15% for fungi), but on the other hand, they are a very rich source of vitamins and minerals (A. G. Pereira, Fraga‐Corral, et al. 2022). One possibility for producing single‐cell protein is the utilization of straw‐based biomass (Z. Zhang et al. 2024). Saccharides are important for fermentation, especially sugar and starch. It also depends on the humidity of the substrate, which is intended for production (Li et al. 2024). In addition to the high protein content, single‐cell proteins also contain significant amounts of vitamins and minerals (Li et al. 2024). According to A. G. Pereira, Fraga‐Corral, et al. (2022), their amino acid profile is similar to that of soybean meal. Gundupalli et al. (2024) compared amino acid profiles of soybean meal and commercial preparations based on single‐cell protein (Uniprotein, KnipBio, StringBio), found that soybean meal had a relatively lower total amino acid content. Furthermore, Gundupalli et al. (2024) noted that the inclusion of single‐cell protein in diets could reduce the use of fishmeal. With a decrease in fishmeal consumption, there would likely be a decrease in the catch of fish used for feed purposes.

#### 2.6.1 Single‐Cell Protein in Poultry Nutrition

Research by Hombegowda et al. (2021) shows that it is suitable to include up to 5% of single‐cell protein in feed mixtures for poultry. Broilers fed a diet containing single‐cell protein had similar weight gains and feed conversion as broilers fed diets based on soy and fishmeal. A higher proportion of single‐cell protein (7.5% or 10%) showed worse results for these parameters, but also with a lower intestinal absorption area. In contrast, the study by Bhunia et al. (2023) reported that proportions higher than 2% negatively affect zootechnical parameters such as weight gain, conversion or feed intake. Single‐cell protein synthesized from potato, banana or tomato substrate by the *Aspergillus flavus* bacterium, when included in the diet, did not affect the feed conversion ratio. However, it should be noted that the inclusion level was very low (below 1%) (Khan et al. 2024). There was no effect of the inclusion of single‐cell protein on the carcass weight of broilers compared to a control diet based on soybean meal (Bhunia et al. 2023).

#### 2.6.2 Single‐Cell Protein in Pig Nutrition

H. Y. Zhang et al. (2013) found that replacing 50% of fish meal with commercial single‐protein preparations, specifically Prosin and Protide, did not lead to a significant decrease in the production performance of weaned pigs in their study. However, it's important to note that these single‐cell protein preparations had lower standardized ileal digestibility of amino acids compared to fish meal. Therefore, when incorporating these alternatives, it is essential to accurately formulate the feed ration to ensure equivalent nutrient supply relative to the replaced ingredient. A lower coefficient of standardized ileal digestibility of crude protein was also found in the study by Wang et al. (2013) when comparing single‐cell protein with soybean meal in growing pigs.

## 3 Conclusions

The growing global population and the resulting increase in protein demand, both directly for human consumption and indirectly for livestock, present new challenges and opportunities for agriculture. However, this increased demand for protein must be balanced against sustainability and environmental considerations. Currently, soy and its by‐products, which represent the primary protein source for non‐ruminant animals, do not meet these requirements. While legumes, such as lupine, peas and faba beans, do not reach the protein content in the same amount as soy, partial replacement should not significantly affect the production performance of non‐ruminant animals. The advantage of pulses compared to soy is the lower consumption of fossil fuels and the production of by‐products due to transport, as pulses are a traditional crop in Europe. However, it is also necessary to indicate the content of anti‐nutritional substances, which necessitates feed processing before feeding. Insects, single‐cell protein and seaweed have a high protein content, which can be even higher than soy, and their production also does not require cutting down rainforests for crop areas. However, it must be recognized that these feeds are expensive to produce, which puts them at a disadvantage compared to soy. Experiments involving these alternative feeds are still at a relatively early stage, and it is therefore desirable to continue research to improve understanding of their effects on animal performance and other production parameters.

The available data indicate that the selected feed sources have the potential to partially replace soy and soy‐derived products in the nutrition of non‐ruminants; however, it is also necessary to account for certain adverse effects associated with their use.

## Conflicts of Interest

The authors declare no conflicts of interest.

## Data Availability

Data sharing is not applicable to this article as no data sets were generated or analysed during the current study.
